# The Evaluation of the Quality Performance of Biochemical Analytes in Clinical Biochemistry Laboratory Using Six Sigma Matrices

**DOI:** 10.7759/cureus.51386

**Published:** 2023-12-31

**Authors:** Chhabi R Panda, Suchitra Kumari, Manaswini Mangaraj, Saurav Nayak

**Affiliations:** 1 Biochemistry, All India Institute of Medical Sciences, Bhubaneswar, IND

**Keywords:** quality control, quality assurance, root cause analysis, quality goal index, six sigma

## Abstract

Introduction

This study was conducted to assess the analytical performance of biochemical tests using Six Sigma methodology and to assess the underlying causes of unsatisfied performance of analytes with a sigma value of less than 4 using quality goal index (QGI) and root cause analysis (RCA).

Methodology

Daily data for internal quality control (IQC) for both level 1 (L1) and level 2 (L2) and monthly data for external quality assessment for a period of six months were recorded. The coefficient of variation (CV), bias, and total allowable error (TEa) were calculated to analyze the sigma (σ) values for 19 biochemical analytes. Quality goal index (QGI) analysis was done to analyze impressions and inaccuracies in analyte performance having a sigma value of less than 4. Root cause analysis (RCA) was done to evaluate the possible causes that can improve quality performance.

Results

Creatinine and high-density lipoprotein (HDL) had sigma metrics of ≤2.0, and chloride, aspartate aminotransferase (AST), and alkaline phosphatase (ALP) had sigma values between 2 and 3. Glucose, total protein (TP), phosphate (Phos), and potassium had sigma values between 4 and 5 in level 1 QC. Sigma grading for level 2 quality control (QC) also gave similar results. For analytes with σ < 4, QGI analysis exposed inaccuracy or imprecision issues and identified errors such as the reconstitution of IQC, storage temperature, and air bubbles while processing the QC, being common causes of poor performance.

Conclusion

Six Sigma approach is helpful for quality assurance and identifying areas for improvement. Assessing Six Sigma metrics should be a routine practice to decide the frequency of QC run and to detect errors in analysis.

## Introduction

Clinical laboratories have a major impact on the medical decision-making process. Generating reliable, reproducible, and timely interpreted test results is the prime responsibility of clinical laboratories. To produce reliable results, laboratories are aimed to adopt the best quality assurance program. Pre-analytical and post-analytical errors are common within the laboratory process system, whereas the analytical phase contains relatively fewer incidences of errors. Even if there is less frequency of analytical errors, strict monitoring of the quality control (QC) of the analytical phase is required [1].

Internal QC (IQC) and external QC (EQC) are often used to assess and maintain the quality of the analytical phase in a clinical chemistry laboratory. It can be evaluated with the aid of the Westgard rules and control charts, such as those made with Levey-Jennings [2]. To verify that the results are accurate enough to be reported, IQC maintains a constant watch over the analytical system. To preserve the correctness of the result that external QC entails evaluating and reporting, the external QC samples are provided by an outside source once a month [3].

Six Sigma is a quality control approach that assesses laboratory process efficiency through statistical computation. Six Sigma was first developed in 1986 by Motorola Company to reduce industrial wastage [4]. In clinical laboratories, Six Sigma was introduced to achieve high quality and near-zero defect rates in healthcare. Through the incorporation of both bias and coefficient of variation (CV) in a laboratory's performance, the sigma offers an objective means of evaluating and comparing laboratory quality. The three parameters that form the basis of sigma analysis are bias, CV%, and total allowable error (TEa) [5]. The total allowable error represents the laboratory's deviation from the true concentration of an analyte from the reported concentration in a patient's sample, which should be within the limit to consider the result as reliable [5]. Bias is the systematic difference between the average value and the true value [6]. The difference between the expected results of the laboratory's test procedure and the results of an approved reference also reveals bias. The references that are commonly used may be a different method for a particular test, or it can be a standard or a consensus method of references such as proficiency, as well as a peer comparison program among the participating laboratories. Bias should be identified and usually be removed from the laboratory test system as it can have a significant impact on analytical quality. Standard deviation (SD) is the total analytical standard deviation of the test method. The number of analytical standard deviations of the test system process that fit within the specified allowable total error limits represents the sigma metric [7]. The aim of Six Sigma is to bring down the defects per million to 3.4, and the sigma value is inversely related to the number of defects [8]. Its evaluation reveals surprising differences in the performance of the analytes in the laboratories and helps in deciding the frequency of QC, and the concentration of different levels of QC required improving the performance of the analytes. The present study evaluated the 19 biochemical parameters' analytical performance by calculating sigma values for each analyte using CV%, bias, and TEa (Clinical Laboratory Improvement Amendment {CLIA} 2024 and Randox International Quality Assessment Scheme {RIQAS} 2021) and analyzed quality goal indices (QGI) for all analytes; also, root cause analysis (RCA) was done for analytes with poor performance.

## Materials and methods

Study setting

This prospective observational study was conducted in the clinical biochemistry laboratory of All India Institute of Medical Sciences (AIIMS), Bhubaneswar.

Data collection

Institutional ethics committee (IEC) permission was taken via the letter number T/IM-NF/Biochem/22/30 dated 07/07/2022 before the commencement of this study. The internal quality control data required for this study were collected from January 2022 to September 2022, at the clinical biochemistry laboratory using daily QC material from Bio-Rad Laboratories, Inc. General quality control level 1 (L1) and level 2 (L2) represent normal concentration and abnormal concentration, respectively. Data for 19 analytes, glucose, urea, creatinine, total bilirubin (TBIL), total protein (TP), albumin (ALB), phosphate (Phos), uric acid (UA), total cholesterol (TC), triglyceride (TG), high-density lipoprotein (HDL), sodium (Na+), potassium (K+), chloride (Cl-), aspartate aminotransferase (AST), alanine aminotransferase (ALT), alkaline phosphatase (ALP), amylase (AMY), and iron, were collected. The quality control was performed on a fully automated chemistry analyzer, Beckman Coulter AU5800 system (Brea, CA). Sodium (Na+), potassium (K+), and chlorine (Cl-) were analyzed using the ion-selective electrode (ISE) module. QC materials were provided by Bio-Rad (third party).

Data for external quality control (EQC) were gathered via External Quality Assurance Services (EQAS) provided by Christian Medical College and Hospital (CMC), Vellore. In the clinical biochemistry laboratory, EQAS samples were analyzed for all the 19 analytes, and their results were sent for external validation on a monthly basis. The EQAS performance reports obtained from CMC, Vellore, were gathered and evaluated along with the IQC data. The total allowable error (TEa) data were obtained from the proficiency testing criteria of the American Clinical Laboratory Improvement Amendment (CLIA) 2024 and RIQAS 2021. Any analyte with nonconformity of an EQAS activity (score < 80%) was not included for analysis [9].

Calculation of coefficient of variation (CV%)

CV is standard deviation (SD) expressed as a percentage and is a measure of the variability of the IQC data and expressed as a percentage. CV was calculated using daily IQC data using the following formula: CV = (SD / mean) × (100).

Calculation of bias

Bias is the systematic difference between the results that would be achieved using a recognized reference method and the predicted results produced by the laboratory test procedure. The CMC-EQAS was used to calculate the bias% for each parameter. The formula used to calculate bias is as follows: Bias% = │Measured value in our laboratory - Determined value given by EQAS│/ (Determined value given by EQAS) × 100%.

Calculation of sigma

Sigma metrics were calculated for each analyte on two levels as follows: Sigma = (TEa - Bias) / CV [10].

Quality goal index (QGI) ratio

The QGI ratio was calculated for parameters with a sigma value of less than 4. The calculation equation is as follows: QGI = Bias% / (1.5 × CV%) [11].

A QGI value of less than 0.8 (QGI < 0.8) indicates imprecision [11]. A value greater than 1.2 (QGI > 1.2) indicates inaccuracy [11]. A QGI value between 0.8 and 1.2 (0.8 ≤ QGI ≤ 1.2) indicates both inaccuracy and imprecision [11]. Values with lower sigma were evaluated for the QGI to see where the problem lies, i.e., precision or accuracy or a combination of both.

Root cause analysis (RCA)

**Figure 1:**
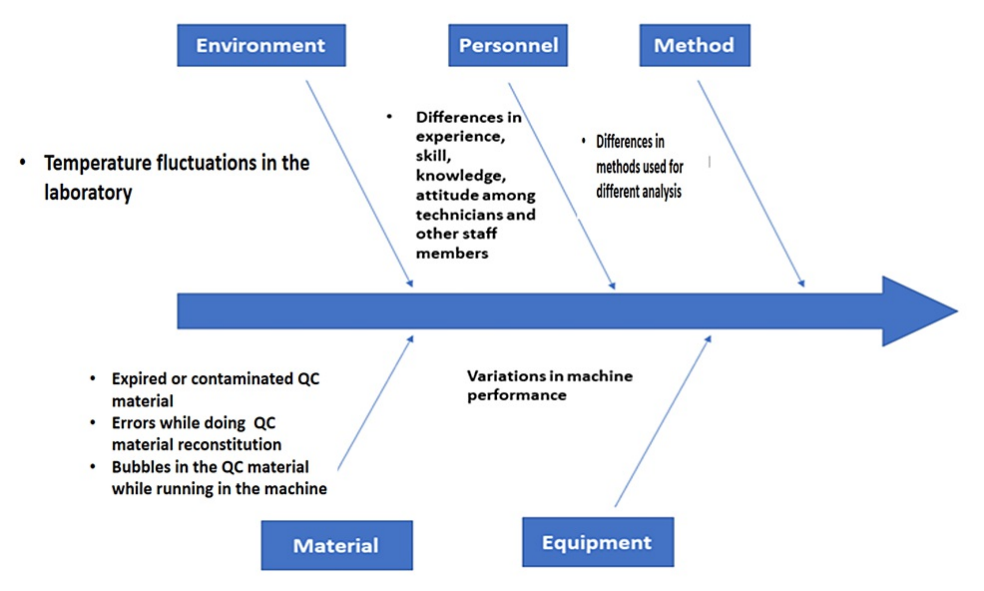
Fishbone diagram for root cause analysis QC: quality control

RCA was performed using a fishbone diagram or cause-effect diagram based on mainly four aspects such as environment, personnel, method, material, and equipment. Figure 1 shows the fishbone diagram.

## Results

Table 1 and Table 2, respectively, display the CV%, bias%, and sigma values of 19 analytes for IQC levels 1 and 2 and all the runs performed on the Beckman Coulter AU5800 auto-analyzer. Values for TEa were extracted from RIQAS SOTA 2021 and CLIA 2024. As indicated in Tables 1, 2, the performance of the analytes was graded into six categories based on the sigma level: world-class (sigma ≥ 6), excellent (5 ≤ sigma < 6), good (4 ≤ sigma < 5), marginal (3 ≤ sigma < 4), poor (2 ≤ sigma < 3), and unacceptable (sigma < 2) [12].

**Table 1:** Sigma metrics for level 1 internal quality control for analytes TEa, total allowable error; CV, coefficient of variation; CLIA, Clinical Laboratory Improvement Amendment; RIQAS, Randox International Quality Assessment Scheme

Serial number	Analytes	TEa	TEa source	Bias	CV	Sigma
1	Glucose	10	CLIA 2024	1.47	1.81	4.72
2	Urea	9	CLIA 2024	0.96	2.32	3.47
3	Creatinine	10	CLIA 2024	0.29	5.14	1.89
4	Total bilirubin	20	CLIA 2024	0.24	3.45	5.73
5	Total protein (TP)	8	CLIA 2024	1.04	1.53	4.54
6	Albumin	10	CLIA 2024	2.10	1.47	5.39
7	Phosphate (Phos)	10	CLIA 2024	0.83	2.21	4.15
8	Uric acid	10	CLIA 2024	0.17	1.20	8.16
9	Total cholesterol (TC)	10	CLIA 2024	2.70	1.03	7.09
10	Triglycerides	15	CLIA 2024	1.09	1.93	7.22
11	High-density lipoprotein (HDL)	10	CLIA 2024	5.01	3.97	1.26
12	Sodium (Na+)	3.6	RIQAS SOTA 2021	5.90	0.76	3.02
13	Potassium (K+)	5.6	RIQAS SOTA 2021	1.80	0.87	4.35
14	Chloride (Cl-)	5	CLIA 2024	2.42	0.91	2.85
15	Aspartate aminotransferase (AST)	15	CLIA 2024	7.04	3.88	2.05
16	Alanine aminotransferase (ALT)	15	CLIA 2024	1.64	2.49	5.37
17	Alkaline phosphatase (ALP)	20	CLIA 2024	9.19	5.15	2.10
18	Amylase	20	CLIA 2024	37.70	3.21	5.51
19	Iron	15	CLIA 2024	3.06	1.17	10.17

**Table 2:** Sigma metrics for level 2 internal quality control for analytes TEa, total allowable error; CV, coefficient of variation; CLIA, Clinical Laboratory Improvement Amendment; RIQAS, Randox International Quality Assessment Scheme

Serial number	Analytes	TEa	TEa source	Bias	CV	Sigma
1	Glucose	10	CLIA 2024	1.47	1.78	4.79
2	Urea	9	CLIA 2024	0.96	1.79	4.48
3	Creatinine	10	CLIA 2024	0.29	3.17	3.07
4	Total bilirubin	20	CLIA 2024	0.24	2.16	9.17
5	Total protein (TP)	8	CLIA 2024	1.04	1.19	5.87
6	Albumin	10	CLIA 2024	2.10	1.66	4.76
7	Phosphate (Phos)	10	CLIA 2024	0.83	1.67	5.51
8	Uric acid (UA)	10	CLIA 2024	0.17	0.83	11.91
9	Total cholesterol (TC)	10	CLIA 2024	2.70	1.20	6.08
10	Triglycerides	15	CLIA 2024	1.09	2.97	4.69
11	High-density lipoprotein (HDL)	10	CLIA 2024	5.01	5.72	0.87
12	Sodium (Na+)	3.6	RIQAS SOTA 2021	5.90	0.70	3.29
13	Potassium (K+)	5.6	RIQAS SOTA 2021	1.80	1.80	2.11
14	Chloride (Cl-)	5	CLIA 2024	2.42	4.10	0.63
15	Aspartate aminotransferase (AST)	15	CLIA 2024	7.04	2.11	3.77
16	Alanine aminotransferase (ALT)	15	CLIA 2024	1.64	8.87	1.51
17	Alkaline phosphatase (ALP)	20	CLIA 2024	9.19	1.35	8.01
18	Amylase	20	CLIA 2024	37.70	2.20	8.03
19	Iron	15	CLIA 2024	3.06	1.75	6.81

Table 3 shows the sigma values in terms of six grades and the analytes falling under those categories for both level 1 and level 2 IQC data. Creatinine and HDL had sigma metrics of ≤2.0, and Cl-, AST, and ALP had sigma values between 2 and 3. Urea and Na+ had σ values between 3 and 4, and glucose, total protein, phosphate, and potassium had sigma values between 4 and 5. Four analytes showed sigma values between 5 and 6, and only four out of 19 analytes, uric acid, total cholesterol, triglyceride, and iron, have achieved sigma values of 6 (Table 3). Similarly, the sigma grading for internal quality control level 2 is also given in Table 3.

**Table 3:** Sigma grades of analytes for internal quality control (IQC) level 1 (L1) and level 2 (L2)

Sigma grades	Analytes (IQC-L1)	Analytes (IQC-L2)
World class (σ ≥ 6)	Uric acid (UA), total cholesterol (TC), triglycerides (TAG), and iron	Total bilirubin (TBIL), uric acid (UA), total cholesterol (TC), alkaline phosphatase (ALP), amylase, and iron
Excellent (5 ≤ σ < 6)	Total bilirubin (TBIL), albumin (ALB), alanine aminotransferase (ALT), and amylase	Total protein (TP) and phosphate (Phos)
Good (4 ≤ σ < 5)	Glucose, total protein (TP), phosphate (Phos), and potassium (K+)	Glucose, urea, albumin (ALB), and triglycerides
Marginal (3 ≤ σ < 4)	Urea and sodium (Na+)	Creatinine (Creat), sodium (Na+), and aspartate aminotransferase (AST)
Poor (2 ≤ σ < 3)	Chloride (Cl-), aspartate aminotransferase (AST), and alkaline phosphatase (ALP)	Potassium (K+)
Unacceptable (σ < 2)	Creatinine (Creat) and high-density lipoprotein (HDL)	High-density lipoprotein (HDL), chloride (Cl-), and alanine aminotransferase (ALT)

**Figure 2:**
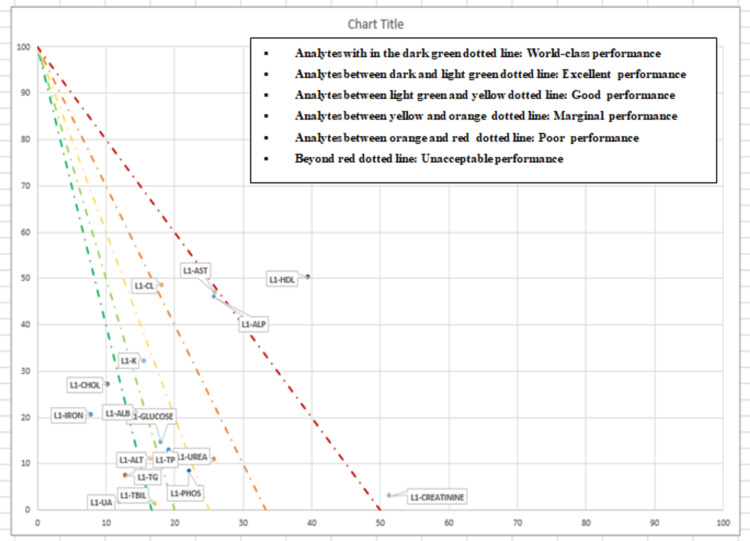
Method decision chart to represent the sigma value of analytes for IQC-L1 X-axis: percentage of observed imprecision (CV/TEa); Y-axis: percentage of observed inaccuracy (bias/TEa) IQC-L1, internal quality control level 1; CV, coefficient of variation; TEa, total allowable error

**Figure 3:**
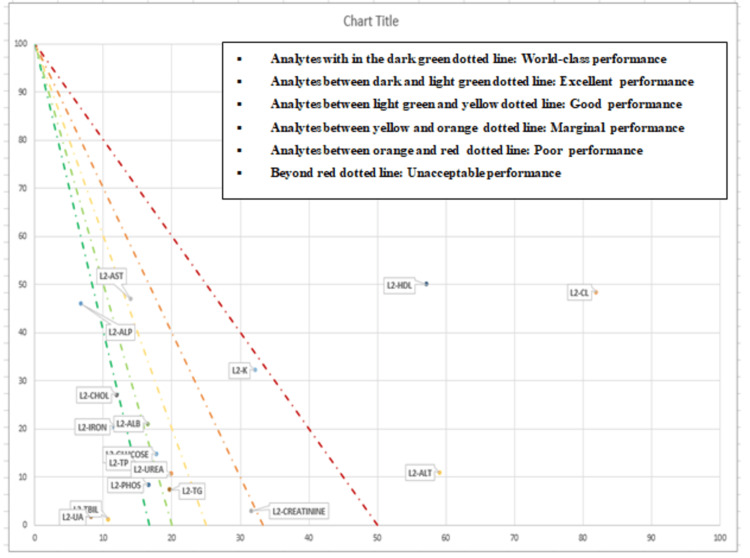
Method decision chart to represent the sigma value of analytes for IQC-L2 X-axis: percentage of observed imprecision (CV/TEa); Y-axis: percentage of observed inaccuracy (bias/TEa) IQC-L2, internal quality control level 2; CV, coefficient of variation; TEa, total allowable error

The sigma value for the analytes was represented in graphical form by method decision chart in Figure 2 and Figure 3 for level 1 and level 2, respectively.

Quality goal index (QGI) was calculated for seven analytes with σ < 4. Table 4 shows QGI values for level 1 IQC runs. It has been seen that out of seven analytes, four analytes have shown problem in precision, and three had problem with accuracy in the results. Similarly, Table 5 shows QGI values and problems for level 2 IQC runs.

**Table 4:** Quality goal index (QGI) of analytes for level 1 (L1) IQC run IQC: internal quality control

Analytes	QGI (L1)	Problem
Urea	0.3	Imprecision
Sodium (Na+)	5.2	Inaccuracy
Chloride (Cl-)	1.8	Inaccuracy
Aspartate aminotransferase (AST)	1.2	Inaccuracy and imprecision
Alkaline phosphatase (ALP)	1.2	Inaccuracy and imprecision
Creatinine (Creat)	0.03	Imprecision
High-density lipoprotein (HDL)	1.26	Inaccuracy

**Table 5:** Quality goal index (QGI) of analytes for level 2 (L2) IQC run IQC: internal quality control

Analytes	QGI (L2)	Problem
Creatinine (Creat)	0.1	Imprecision
Sodium (Na+)	5.6	Inaccuracy
Aspartate aminotransferase (AST)	2.2	Inaccuracy
Potassium (K+)	0.7	Imprecision
High-density lipoprotein (HDL)	0.6	Imprecision
Chloride (Cl-)	0.4	Imprecision
Alanine aminotransferase (ALT)	0.1	Imprecision

Root cause analysis was done based on five potential factors that can lead to low performance of analytes and low sigma value: environmental factors, personnel factors, method followed for different analytes, materials used for both L1 and L2 IQC, and finally the equipment-related errors. These factors were represented on the fishbone analysis chart (Figure 1). Errors while reconstituting IQC, storage temperature, and air bubbles while processing the QC were more common causes of poor performance.

## Discussion

The application of the Six Sigma methodology within the context of clinical biochemistry laboratory analytical performance has yielded significant improvements [11]. The conventional quality control measurement can only monitor daily performance but is unable to indicate unstable performances. The Westgard sigma Rules provide a thorough evaluation of the performance of the analytes, and based on that the parameters, low sigma quality could be rectified by proper quality control rules and more frequent quality control measurements. Six Sigma metrics detect medically important errors and provide a simple and quick way of selecting control rules and the number of control measurements needed [13]. The application of Six Sigma principles can also lead to a notable reduction in costs associated with reagents, equipment maintenance, and personnel time [7]. The decreased frequency of errors and rework not only saves valuable resources but also reduces the potential for misdiagnoses and subsequent costly treatments. This cost reduction is particularly significant in the context of resource-constrained healthcare environments.

The final sigma value may be affected by the various methods used to calculate bias and CV. For instance, bias could be detected using internal quality control data or external QC data. In the present study, we used internal quality control data and analyzed the performance of 19 biochemical analytes using Six Sigma. Out of 19 analytes, UA, TC, TG, and iron were found to show world-class performance with a sigma value of more than 6 with level 1 QC material, and TBIL, UA, TC, ALP, amylase, and iron have shown similar result for level 2 QC material. The worst analytical performance with a sigma value of less than 2 was shown by Creat and HDL and HDL, Cl-, and ALT for IQC levels 1 and 2, respectively. It was seen that the main reason for the low sigma value for analytes that showed unacceptable analytical performance was impression, which was in turn due to variation in IQC data. Changes in reagents, calibrators, or staff could have an impact on the CV, a measure of imprecision that was based on IQC data over a six-month period in our study. The current CV is anticipated to be wider than the CV calculated over shorter time periods, which could have resulted in lower sigma metrics. van Heerden et al. reported that sigma also varies as per total allowable error [8].

In order to understand the underlying cause, we calculated the QGI for analytes with a sigma value of less than 4, and it was seen that imprecision was the most common reason for a low sigma value. QGI furnished robust instructions to address the issues related to the analytes' performance, such as imprecision or inaccuracy. In the present study, to verify the precision, five observations in a reference material were recorded over five days, and a root cause analysis was done, to look for factors such as environmental; personnel, i.e., random error or procedure/method; and instrument, systematic error or bias. As per the study by Zhou et al. for both levels, sigma method decision charts have been generated, which combined all of the sigma metrics into an easy-to-understand dashboard [11]. Analytes were categorized as world-class, excellent, good, marginal, poor, and unacceptable based on their sigma values. The integration of Six Sigma methodology into clinical biochemistry laboratories has been extensively explored in previous studies [11,12]. The review by Westgard et al. demonstrated that Six Sigma metrics provide a robust framework to analyze laboratory errors and optimize the QC rules as per the performance of the laboratory tests [14]. This corroborates our findings, which highlight the effectiveness of Six Sigma in objectively assessing the analytical performance of the laboratory.

While QGI helps in determining the cause for lower sigma metrics, i.e., imprecision and inaccuracy, root cause analysis (RCA) can help to take necessary steps to deal with shortcomings found in QGI [11]. In the present study, errors while reconstituting IQC, storage temperature, and air bubbles while processing the QC were more common, suggesting the fact that quality control management with a dedicated staff plays a paramount role in managing the performance of the biochemical parameters. The practice of Six Sigma had helped us in understanding the performance of common biochemical parameters, detecting the lapses in routine laboratory practice, and providing a direction/scope for improvement in laboratory management. This was a cross-sectional study evaluating the sigma metrics of biochemical parameters.

The limitation of this study is that the performance following the corrective measures could not be evaluated prospectively. A follow-up study with corrective measures will further substantiate the usefulness of Six Sigma in analytical performances. Additionally, future studies focusing on integrating advanced analytical technologies and artificial intelligence algorithms within the Six Sigma framework will further optimize analytical performance.

## Conclusions

The application of Six Sigma methodology in the clinical biochemistry laboratory setting has demonstrated significant potential for improving analytical performance. It helps in detecting the lapses in routine laboratory practice and provides scope for corrective measures. The tangible benefits in terms of accuracy, efficiency, cost reduction, and employee engagement underscore the value of this approach in enhancing the quality of healthcare delivery. By addressing the identified challenges and embracing opportunities for further refinement, Six Sigma can continue to play a pivotal role in advancing clinical laboratory practice.
